# Legacies from early‐season hot drought: how growth cessation alters tree water dynamics and modifies stress responses in Scots pine

**DOI:** 10.1111/plb.13760

**Published:** 2025-01-15

**Authors:** N. K. Ruehr, D. Nadal‐Sala

**Affiliations:** ^1^ Institute of Meteorology and Climate Research (IMK‐IFU), KIT‐Campus Alpin Karlsruhe Institute of Technology (KIT) Garmisch‐Partenkirchen Germany; ^2^ Centre de Recerca Ecològica i Aplicacions Forestals (CREAF) Campus de Bellaterra (UAB) Edifici C Cerdanyola del Vallès Spain; ^3^ Ecology Section Universitat de Barcelona (UB) Barcelona Spain

**Keywords:** Drought legacy, drought timing, needle elongation, seasonal drought, structural adjustment, tree growth, tree water deficit

## Abstract

- Tree responses to drought are well studied, but the interacting effects of drought timing on growth, water use, and stress legacy are less understood. We investigated how a widespread conifer, Scots pine, responded to hot droughts early or late in the growing season, or to both.
- We measured sap flux, stem growth, needle elongation, and leaf water potential (*Ψ*_leaf_) to assess the impacts of stress timing on drought resilience in Scots pine saplings.
- The early summer hot drought had peak temperatures of 36.5 °C, while the late summer hot drought peaked at 38.2 °C. Soil water content during both periods declined to ca. 50% of control values. The early‐season hot drought caused growth cessation already at *Ψ*_leaf_ − 1.1 MPa, visible as an almost 30 days earlier end to needle elongation, resulting in needles 2.7 cm shorter, on average. This reduction in leaf area decreased productivity, resulting in a reduction of 50% in seasonal transpiration. However, the reduced water use of early‐stressed saplings appeared to enhance resistance to a late‐season drought, as reflected in a smaller decline in *Ψ*_leaf_ and lower tree water deficit compared to saplings that did not experience early‐season stress.
- In summary, we observed persistant drought legacy effects from early‐season hot‐drought stress, as evident in a 35% reduction of leaf area, which impacted tree water use, stress resistance, and productivity. These structural adjustments of leaf development and reduced bud mass from early‐season stress could be critical in evergreen conifers, whose long‐lived foliage influences future water use and growth potential.

## Graphical abstract

Early‐season hot drought in Scots pine limits growth and productivity but improves resilience to late‐season drought through reduced water use.

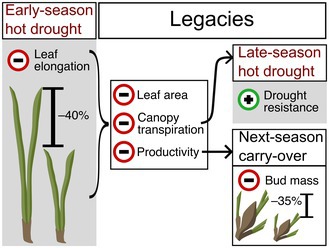

## Introduction

The frequency and intensity of hotter droughts is increasing in many regions globally (Overpeck 2013; Ault 2020; Markonis *et al*. 2021). This trend has significant yet not fully understood implications for the carbon (C) and water cycles of Earth's forests. In Central Europe, summer drought periods accompanied by high temperatures and increased evaporative demand have become more frequent over recent decades (Markonis *et al*. 2021), with considerable variability in drought duration, intensity, and timing. The devastating 2018 European summer drought developed early in the season, alongside high temperatures, resulting in high tree mortality (Schwarz *et al*. 2023), and had more negative impacts on forest functioning than the previous benchmark drought of 2003, *e.g*., visible as decreases in vegetation greenness (Schuldt *et al*. 2020). The primary difference between these two extreme events is the timing of peak drought, which occurred earlier in 2018 compared to 2003, together with higher temperatures (Buras *et al*. 2020). Furthermore, with ongoing climate warming, the summer period has extended, with more hot days, increasing the likelihood of new temperature extremes and rises in vapor pressure deficit (VPD) early in the season when newly developed tissues are highly sensitive to stress damage (Misson *et al*. 2011; Adams *et al*. 2015; D'Orangeville *et al*. 2018; Grossman 2023). A shift in the timing of stress from later to earlier in the growing season could have longer‐term consequences for forest C uptake and tree growth, but this has received little attention so far.

Tree growth is highly water‐sensitive, as cell division and expansion are turgor‐dependent (Lockhart 1965). Even moderate reductions in water potential can limit leaf growth (Tang & Boyer 2002), and cambium cell differentiation (Körner 2015). Increasing evidence suggests that plant growth is more sensitive to water limitation than photosynthesis, stopping well before stomatal closure (Muller *et al*. 2011). This implies that water limitation, whether induced by soil drought or atmospheric drought (VPD), can significantly control tree structural growth and C allocation. Moreover, increases in VPD exacerbate tree water stress, especially under mild to moderate drought (Ruehr *et al*. 2016), limiting C uptake and thus tree growth (Zhao *et al*. 2013; Novick *et al*. 2016; Sanginés de Cárcer *et al*. 2018). For instance, a significant role of VPD in limiting stem growth in temperate conifers and broadleaf trees has been demonstrated under both non‐drought and moderate drought conditions, with conifers generally being more sensitive to soil drought (Zweifel *et al*. 2021). This indicates that even mild water limitation can affect tree growth, potentially leading to longer‐term consequences for C storage.

Tree growth responses to soil and atmospheric drought vary both temporally and seasonally (Peltier & Ogle 2020). For instance, wood formation in temperate forests has been shown to be more strongly limited by spring droughts than by summer droughts (D'Orangeville *et al*. 2018). Consistent findings were reported for 20 conifer species in a common garden experiment, where approximately half exhibited reduced growth when drought occurred earlier in the growing season (Song *et al*. 2022). Similarly, tree rings in two dominant oak species showed that spring droughts reduced subsequent growth during recovery more significantly than later summer droughts (Bose *et al*. 2021). In contrast, other studies have found a more pronounced drought legacy when droughts occur later in the season (Huang *et al*. 2018; Kannenberg *et al*. 2019). This phenomenon has been attributed to a larger impact on C reserve formation, which limits tree ring growth in the following year. While it is clear that timing of drought stress affects woody growth, the magnitude of the response, particularly the ability of trees to recover post‐stress, remains elusive (Ruehr *et al*. 2019; Kannenberg *et al*. 2020). Moreover, different tissues and growth processes may have varying sensitivities to drought (Hsiao & Xu 2000), adding further complexity to understanding tree dynamics under differently timed stress.

Further, stress vulnerabilities of tissues and organs can change seasonally. For instance, newly developed leaf tissues are most susceptible to drought (Herrera *et al*. 2022) and high temperatures (Ruehr *et al*. 2016) early in the season, while their susceptibility typically decreases as the season progresses (Grossman 2023). This may be related to lower leaf thermotolerance early in the season (Grossman 2023) or increased drought tolerance as leaves mature, possibly because of a decline in the turgor loss point (Herrera *et al*. 2022) and an increased cuticular barrier (Pantin *et al*. 2012). As a result, early‐season stress may have a larger, more detrimental impact than stress occurring later in the season, leaving trees weaker and less resistant to subsequent droughts. In addition, a drought occurring early in the season could have direct impacts on leaf phenology and development. Few experimental studies have captured stress phenology in trees, as most studies are conducted later in the summer, when primary growth processes are complete. However, studies that experimentally induced early‐season drought stress have found distinct phenological responses, including delayed bud break and reduced leaf development (Misson *et al*. 2011; Adams *et al*. 2015; Gebauer *et al*. 2020), resulting in an overall reduction in leaf area.

Reductions in leaf area triggered by drought either through lower leaf production or leaf senescence can directly reduce water use at the whole‐tree level. For instance, drought‐induced leaf senescence can be advantageous in reducing residual cuticular transpiration during hotter droughts, delaying water potential decline and hydraulic failure, as found for Scots pine saplings (Nadal‐Sala *et al*. 2021). Furthermore, such reductions in leaf area may ultimately provide a buffer against recurrent droughts (Hochberg *et al*. 2017). Particularly in evergreen conifers—because of their long leaf lifespan—these adjustments easily carry over into the next year and may limit both water loss and C uptake (Zweifel *et al*. 2020). In agreement, lagged effects on tree growth have been shown to be more pronounced in gymnosperms than angiosperms (Anderegg *et al*. 2015; Hesse *et al*. 2022), thus increasing the risk of mortality with leaf lifespan in conifers (Sterck *et al*. 2024). Hence, defoliation in Scots pine led to reduced tree growth and a general decline in vigor (Salmon *et al*. 2015). Moreover, stress‐induced reductions in leaf area can significantly delay the refilling of depleted carbohydrate reserves once the stress is relieved. This delay may increase vulnerability to future drought periods, as found for Scots pine (Poyatos *et al*. 2013). Reduced stress resilience of growth has been attributed to increased tree mortality in 20 conifer species (Sterck *et al*. 2024). Hence, reductions in leaf area—either from leaf senescence or reduced leaf production—are potential water‐saving mechanisms at the expense of C uptake. In the short term, they may help maintain tree hydraulic integrity (Nadal‐Sala *et al*. 2021), but in the longer term, they could be early warning signs of tree dieback due to reduced tree vigor (Sterck *et al*. 2024) and competitiveness, or increased vulnerability to pests (Sangüesa‐Barreda *et al*. 2023).

Here, we investigated how saplings of Scots pine (*Pinus sylvestris* L.), a conifer widely distributed in temperate and boreal forest zones with distinct growth phenology, respond to the seasonal timing of hot‐drought stress. Scots pines exhibit high plasticity in growth under stress, typically decreasing leaf area in drier conditions (Mencuccini & Grace 1995). However, compensatory growth following the release of drought stress has also been observed (Seidel *et al*. 2019). To further understand these variable growth processes and potential drought‐induced morphological adjustments, we exposed saplings to hot‐drought stress either early in the leaf development phase, later in the season when leaf development was complete, or during both periods. We compared differences in needle elongation and woody growth responses between early‐season and late‐season hot‐drought stress and examined how these responses influenced seasonal water use. Our key hypotheses were: (i) early‐season stress during leaf expansion has a strong impact on growth which cannot be compensated for later in the season; (ii) stress‐induced leaf area adjustments reduce water loss and mitigate the impact of a late‐season hot drought; and (iii) structural adjustments might potentially induce drought legacies that carry over into the next season.

## MATERIAL AND METHODS

### Experimental set‐up

*Pinus sylvestris* seedlings (Provenance 85 115, Franconia, Germany) were obtained from a local tree nursery in spring 2017. The seedlings were grown in individual 5 L pots outside a scientific greenhouse facility in Garmisch‐Partenkirchen, Germany (708 m a.s.l., 47°28′32.9′′N, 11°3′44.2′′E) and watered regularly during the growing season. The seedlings were re‐potted in November 2018. In order to prepare for the following year's seasonal stress experiment and to allow unrestricted root growth, each of the 3.5‐year‐old Scots pine saplings was transferred into a separate large pot (ca. 120 L; 55 cm upper diameter, 70 cm height). The substrate was a mixture of commercially available potting substrate (pH = 5.5, N: 210 mg L^−1^, P_2_O_5_: 150 mg L^−1^, K_2_O: 270 mg L^−1^, Mg: 100 mg L^−1^; Klasmann‐Deilmann, Geeste, Germany) mixed with perlite and sand in a ratio of 6:3:1, supplemented with 100 g slow‐release fertilizer (Osmocote® Exact Standard 5‐6 M 15–9‐12 + 2MgO + TE, ICL Specialty Fertilizers, Heerlen, The Netherlands). The pots were filled with the substrate mixture to a height of ca. 65 cm. To allow for improved drainage, the bottom layer was filled with a 5‐cm layer of expanded clay. In total, 32 seedlings were planted and randomly assigned to control or one of the three stress treatments: early‐season hot drought (during leaf elongation), late‐season hot drought (end of summer), or both stress periods early‐ and late‐season hot drought (n = 8 per treatment).

**Fig. 1:**
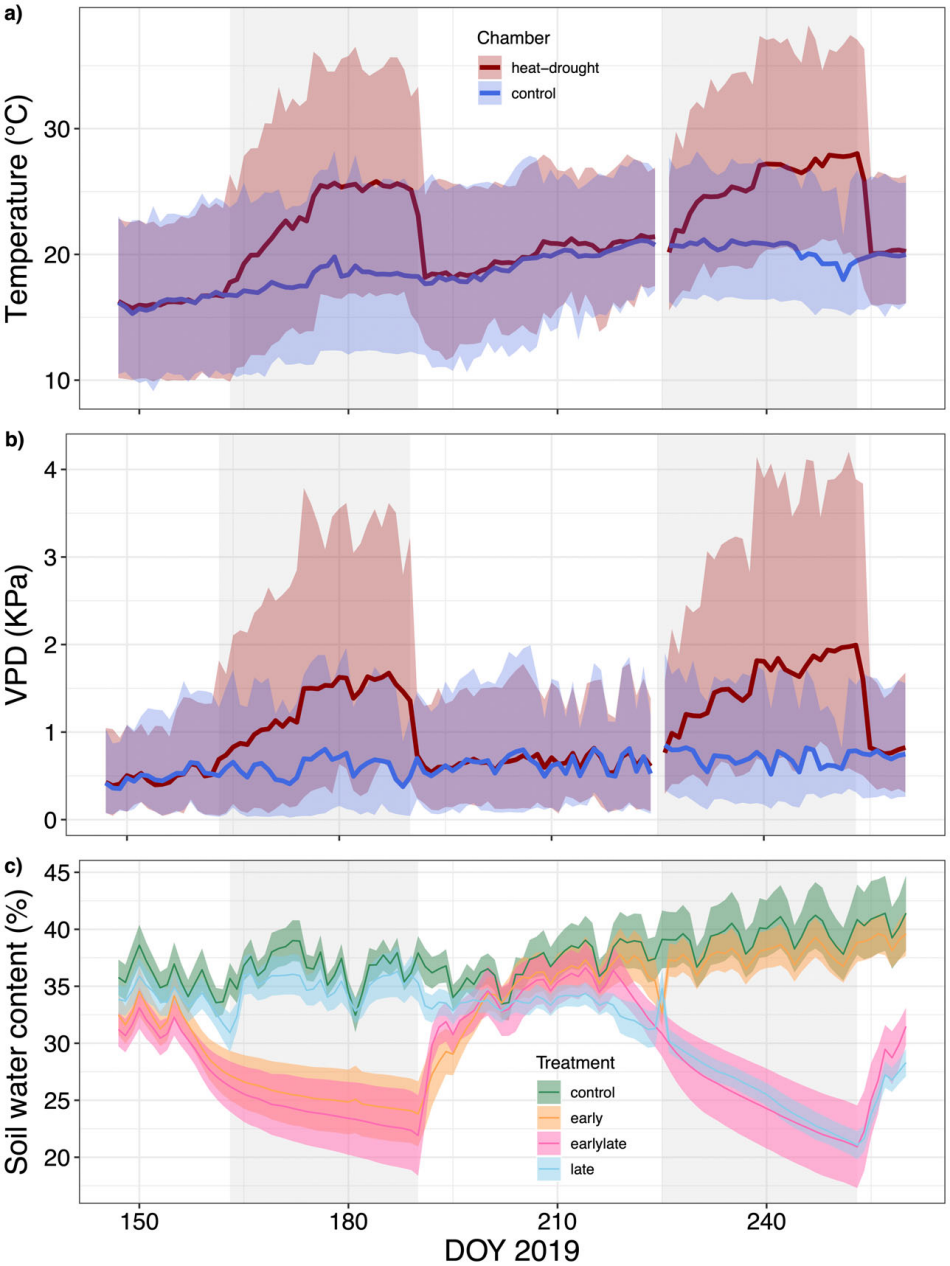
Time series of experimental conditions including the two hot drought periods. Air temperature (a), vapor pressure deficit (b), and soil water content (c) are shown. For air temperature and VPD, the daily amplitude (shaded areas) and daily mean (solid lines) are given per greenhouse compartment. SWC (10–50 cm soil depth) is given as treatment average ± 1SE (shaded area). The two hot drought periods are depicted in the grey shaded areas.

Following potting, the saplings were kept outside during winter. In April 2019, the then 4‐year‐old saplings were moved inside a climate‐controlled greenhouse. In order to allow for higher temperatures and VPD during the stress periods, we placed the saplings in two different greenhouse compartments next to each other. These two adjacent compartments are individually controllable at high precision (Ruehr *et al*. 2016) and referred in the following as ‘ambient’ or ‘stress’ compartment. The saplings in the ambient compartment were initially assigned to control or late stress treatment, and the saplings in the stress compartment were assigned to early or early‐late treatment. We adjusted the environmental conditions in the greenhouse based on long‐term temperature and relative humidity measurements for Franconia, Air temperature during hot drought periods largely reflected conditions during July and August 2003 and 2015 hot and dry summers (see Figure S1). After the hot drought period was initiated, air temperature was stepwise increased by 2 °C (day) and 1.0–1.5 °C (night) every 2–3 days until the maxima experimental temperatures were reached. Apart from the stress periods, the experimental conditions were similar between the two compartments, with differences in air temperature <0.5 °C and VPD <0.02 kPa. In the ambient compartment, mean air temperature was 18.0 °C (20.3 °C) and VPD 1.29 kPa (1.56 kPa) on average during the first (second) experimental stress period. During the first (second) stress period air temperature was on average 5.3 °C (5.4 °C) higher than in the ambient compartment, with average maximum temperatures of 32.7 °C (34.2 °C) (Fig. 1a,b). Before the second stress period, we exchanged placement of the late‐ and early‐treatment saplings between the two greenhouse compartments.

All saplings were watered every 2–4 days to maintain a volumetric soil water content of 35–40% (Fig. 1c). In order to simulate soil drought, watering was completely stopped during the stress periods in the respective treatments. At the end of the stress periods, the stressed saplings were watered with 5–10 l each to allow rapid recovery of the soil water content.

### Continuous measurements

In each of the two individually controlled greenhouse compartments we recorded air temperature, relative humidity and photosynthetically active radiation (PAR) at half‐hour intervals. The PAR sensors (PQS 1; Kipp & Zonen, Delft, The Netherlands) were positioned above the canopy height. Temperature and relative humidity sensors (CS215; Campbell Scientific, Logan, UT, USA) were placed ca. 0.5 m above the vegetation and enclosed in aspirated radiation shields (43502; Young, MI, Traverse City, USA). Additionally, all pots were equipped with soil water content sensors (10HS, Meter, Munich, Germany) at 10 cm below the soil (n = 8 per treatment) and 50 cm soil depth (n = 4 per treatment), and the raw mV signal recorded. To derive average SWC from both soil depths, a substrate‐specific calibration was applied.

To continuously monitor tree growth, water deficit, and canopy transpiration, we installed either high‐resolution point dendrometers (n = 4 per treatment) or sap flux sensors (n = 4 per treatment) on the stems of each sapling. Dendrometer and sap flux sensor were installed on separate trees due to limited stem area. One control tree, which had abnormal diameter growth patterns due to disease, was excluded from the subsequent analyses.

Point dendrometers (DD‐S; Ecomatik, Dachau, Germany; accuracy ±1.5 μm) were installed at a stem height of 5–10 cm. The outermost dead layer of the bark was slightly removed to minimize hygroscopic swelling. Stem diameter variations are attributed to growth and water‐related shrinking or swelling of elastic bark tissues. To estimate water dynamics from dendrometer data, the growth signal was subtracted (see below). We also derived stem basal area variations by adding dendrometer changes to the initial basal area calculated from diameter measurements before sensor installation.

Tree‐level transpiration (E_tree_) was assessed with a heat‐balance sap flow sensor system (EMS 62; EMS Brno, Czech Republic) connected to data loggers (Mini32; EMS). Each sensor, consisting of an unheated and a heated probe including heating elements, was installed at ca. 10 cm stem height below any major branches. To reduce temperature fluctuations, sensors were additionally shielded with aluminum bubble wrap. The temperature difference between heated and unheated probes was set to 4 K. To derive actual sap flow from the measurements, the difference between the heated and unheated probe at zero sap flux velocity was subtracted. Because nocturnal sap flow occurs in most tree species, especially during warm nights, we assumed nighttime transpiration was zero only on nights with an average VPD <0.2 kPa, and linearly interpolated zero‐flow between these nights. The raw signal from each sensor was corrected to an unbiased baseline sap flow signal using the “baseline” package (Liland *et al*. 2010).

All sensors (except sap flow) were measured half‐hourly using data loggers (CR1000; Campbell Scientific, Logan, UT, USA), either directly connected to a logger or via a multiplexer (AM16/32B multiplexer; Campbell Scientific).

### Leaf water potential

Leaf water potential (*Ψ*_leaf_) was measured between 12:00 and 14:00 h. For this we sampled one needle fascicle per tree (n = 8 per treatment) and carefully recut the fascicle at the needle sheet without disturbing the resin ducts. As current‐year needles were still emerging at the beginning of the experiment and not yet dominating the canopy area, 1‐year‐old needles were initially sampled. We switched to current‐year needles after needles had fully emerged and hardening was visually completed by August. The prepared needle fascicle was immediately inserted into the lid of a pressure chamber (Model 1000; PMS Instruments, Albany, OR, USA), which was tightly sealed and slowly pressurized. As soon as water appeared at the cut surface of the fascicle, the pressure reading was taken.

### Needle growth, leaf area, and biomass

Elongation of current‐year needles was monitored weekly following bud burst at the end of April. On each tree, we selected one needle fascicle in the upper tree crown, which we marked to follow development of this needle throughout the experiment. At each measurement campaign, the length of the two needles (n = 8 per treatment) was recorded.

Total organ‐specific biomass was sampled in four saplings per treatment at the end of October. At least two of the sampled saplings per treatment had been equipped with sap flux sensors, the remaining saplings were kept for another experiment. Fresh biomass was separated into buds, current‐year needles, and needles developed before 2019, aboveground woody biomass, and roots. The organ‐specific biomass was dried for 48 h at 60 °C and dry weight recorded. In order to acquire the current and previous year leaf area per sapling from needle biomass, we assessed the specific leaf area (SLA). we sampled three fascicles per tree of current and previous year foliage, determined the year‐specific needle area (Li‐3100; Licor, Lincoln, NB, USA), dried the samples, and recorded the dry weight.

### Data analyses and statistics

Data processing and statistical analyses were performed in R v 4.3.1 (R Core Team 2021).

#### Needle expansion and tree biomass

To analyze needle expansion, we used a truncated linear regression model for each treatment using Bayesian inversion. We modeled needle length as a function of day of the year (DOY), with the model expressed as:

(1) $$L_{\left(\mathrm{DOY},i\right)}=f{\left({\mathrm{DOY}}_{i}\right)}_{1}+f{\left({\mathrm{DOY}}_{i}\right)}_{2}$$

where $f{\left({\mathrm{DOY}}_{i}\right)}_{1}$ denotes increase in needle length up to a specific threshold day, and $f{\left({\mathrm{DOY}}_{i}\right)}_{2}$. captures the expansion trend after the Threshold. These functions are defined as:

(2) $$f{\left({\mathrm{DOY}}_{i}\right)}_{1}=\left\{\begin{array}{c} b_{1}\cdot {\mathrm{DOY}}_{i},\;{\mathrm{DOY}}_{i}\leq \text{Threshold}\\ b_{1}\cdot \text{Threshold},\;{\mathrm{DOY}}_{i}>\text{Threshold} \end{array}\right.$$

(3) $$f{\left({\mathrm{DOY}}_{i}\right)}_{2}=\left\{\begin{array}{c} 0,{\mathrm{DOY}}_{i}\leq \text{Threshold}\\ b_{2}\cdot \left({\mathrm{DOY}}_{i}-\text{Threshold}\;\right),{\mathrm{DOY}}_{i}>\text{Threshold} \end{array}\right.$$

Here, *b*_1_ and *b*_2_ are the daily needle elongation rates (mm day^−1^) before and after the Threshold, and the Threshold is the day when the growth trend changes.

We used broad, uniform priors and ran a 50,000‐iteration Markov‐Chain Monte Carlo simulation with a Gaussian likelihood using the package BayesianTools (Hartig *et al*. 2023). The first 40,000 iterations were discarded as burn‐in, and convergence was confirmed with Gelman‐Rubin scores <1.1. Results are presented as median values with 95% credible intervals (CI), with differences in the Threshold parameter indicating when needle expansion stopped, and non‐overlapping CIs depict significant treatment effects.

Differences among treatments in surface area per needle, bud mass, leaf biomass, root biomass, woody biomass, and leaf area per root biomass were assessed by ANOVA, followed by a post‐hoc Tukey HSD test to determine significant differences among treatments (*P* < 0.05). Bud mass was correlated with leaf area at the end of the experiment using an ANCOVA linear model. Differences in *Ψ*_leaf_ among the stress treatments were compared per measurement campaign using ANOVA followed by a post‐hoc Tukey HSD test.

#### Stem expansion and tree water deficit

To derive tree water deficit and growth patterns for each tree, we detrended the half‐hourly dendrometer signal following the approach outlined by Zweifel *et al*. (2016) and Knüsel *et al*. (2021) using the treenetproc package (R package v 0.1.4). Growth is provided as net diameter increment, which was calculated from daily diameter increments or set to zero if the diameter on any given day did not exceed the previously recorded maximum diameter. Note that tree water deficit (TWD, in mm^2^ day^−1^) is given for basal area to account for potential effects of stem size on diameter shrinkage.

#### Bootstrap analysis

Because of the limited sample size, differences between the treatments in relation to the control were assessed using bootstrap analysis. This was conducted for the following parameters: (i) stem diameter growth, (ii) tree water deficit, (iii) differences in seasonal transpiration, and (iv) proportion of 1‐year‐old needles to total leaf area. For each parameter, samples within each treatment group were obtained by resampling with replacement. The mean values of these resampled groups were then compared to a resampling of the control treatment, which were also obtained by resampling with replacement. This procedure was repeated for 1,000 iterations. We then analyzed the distribution of these differences to determine if they were statistically significant. Significance was established if 95% of the differences did not include zero when compared to the control conditions.

## Results

### Impacts of the seasonal timing of stress on tree growth

**Fig. 2:**
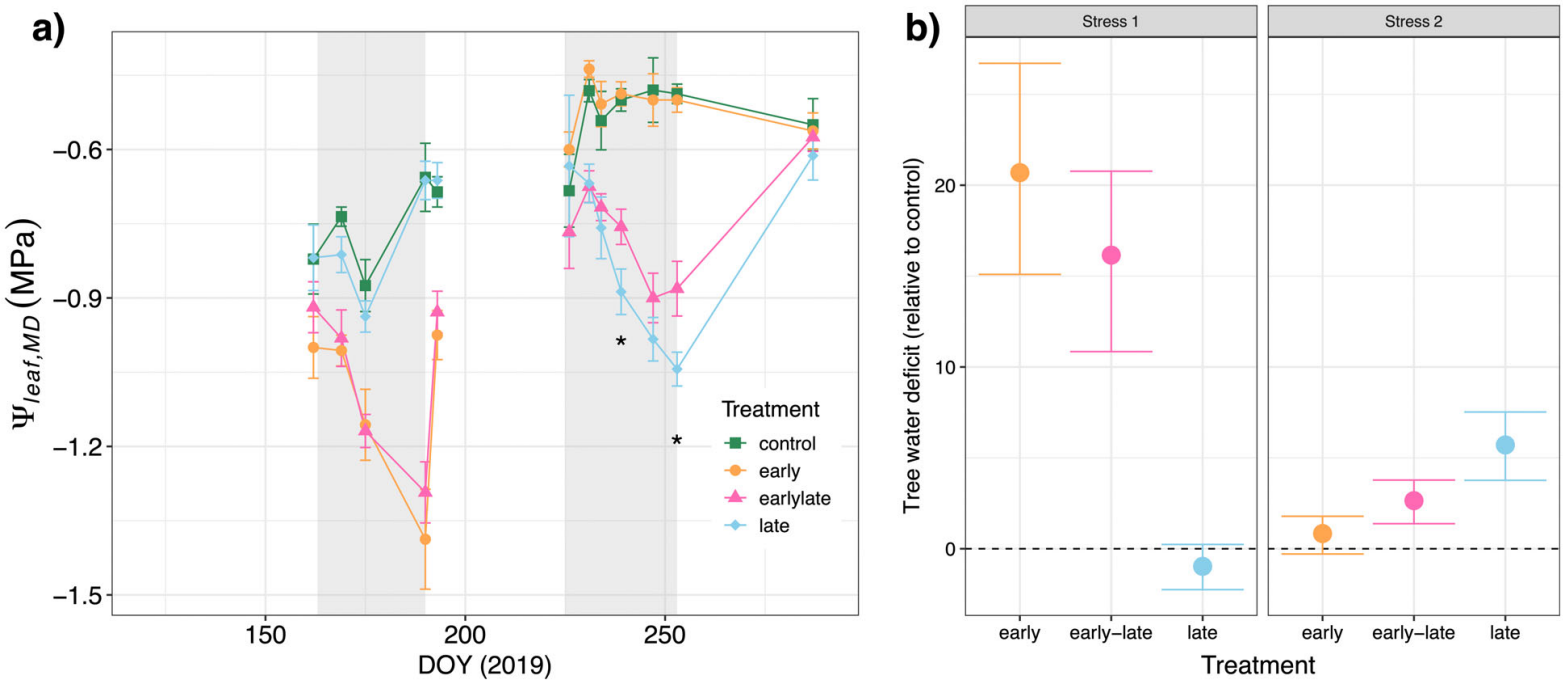
Dynamics of treatment‐specific midday leaf water potential (*Ψ*_leaf_) and relative tree water deficit in Scots pine saplings during two drought phases. (a) Treatment averages ±1SE from 12 sampling campaigns. One‐year‐old needles were measured during the first five campaigns, current‐year needles after full leaf development from August onwards. The switch between needle age classes is indicated by the broken line. The two drought periods are depicted in grey shaded areas. Differences between the stress treatments were tested using ANOVA followed by a post‐hoc Tukey HSD. Significant differences (*P* < 0.05) are highlighted with the asterisk. (b) Relative tree water deficit ± CI derived from stem basal area shrinkage at the peak of drought intensity compared to the control, obtained via bootstrapping. Maximum drought intensity was on DOY 189 during the first (early season) stress period and on DOY 251 during the second (late season) stress period. A significant difference is indicated with non‐overlapping CI, and zero difference from control is given as a broken line.

**Fig. 3:**
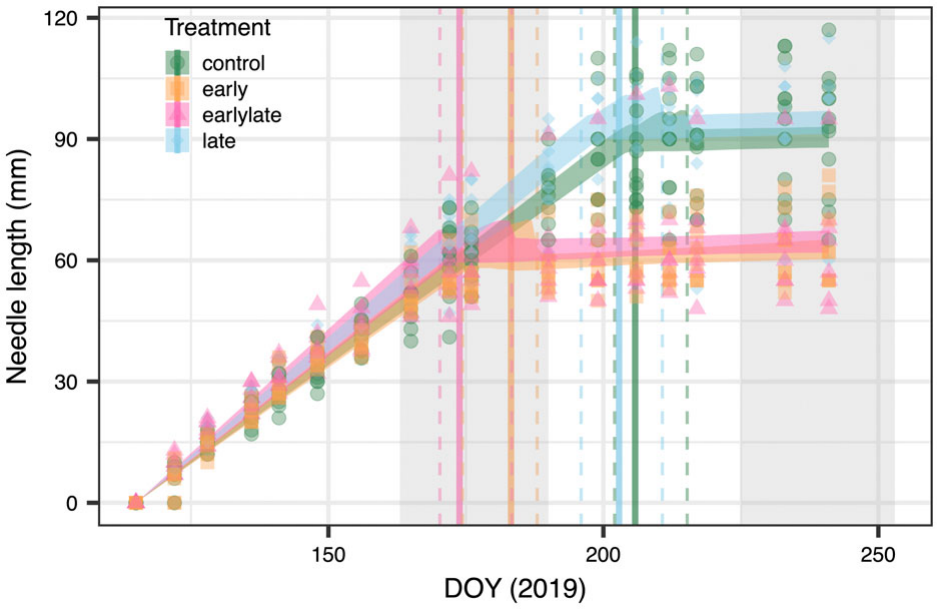
Cumulative needle growth per treatment for Scots pine saplings during two differently‐timed drought periods. Each data point represents an individual measurement. The trend in needle length for each treatment was determined using linear truncated regressions, with the median and 95% CI depicted with shaded lines. Changes in needle expansion trends (see equations (1), (2), (3)) are shown for each treatment with vertical lines, representing the median (solid lines) and the 95% CI (dashed lines). The two hot drought periods are indicated as grey shaded areas.

**Fig. 4:**
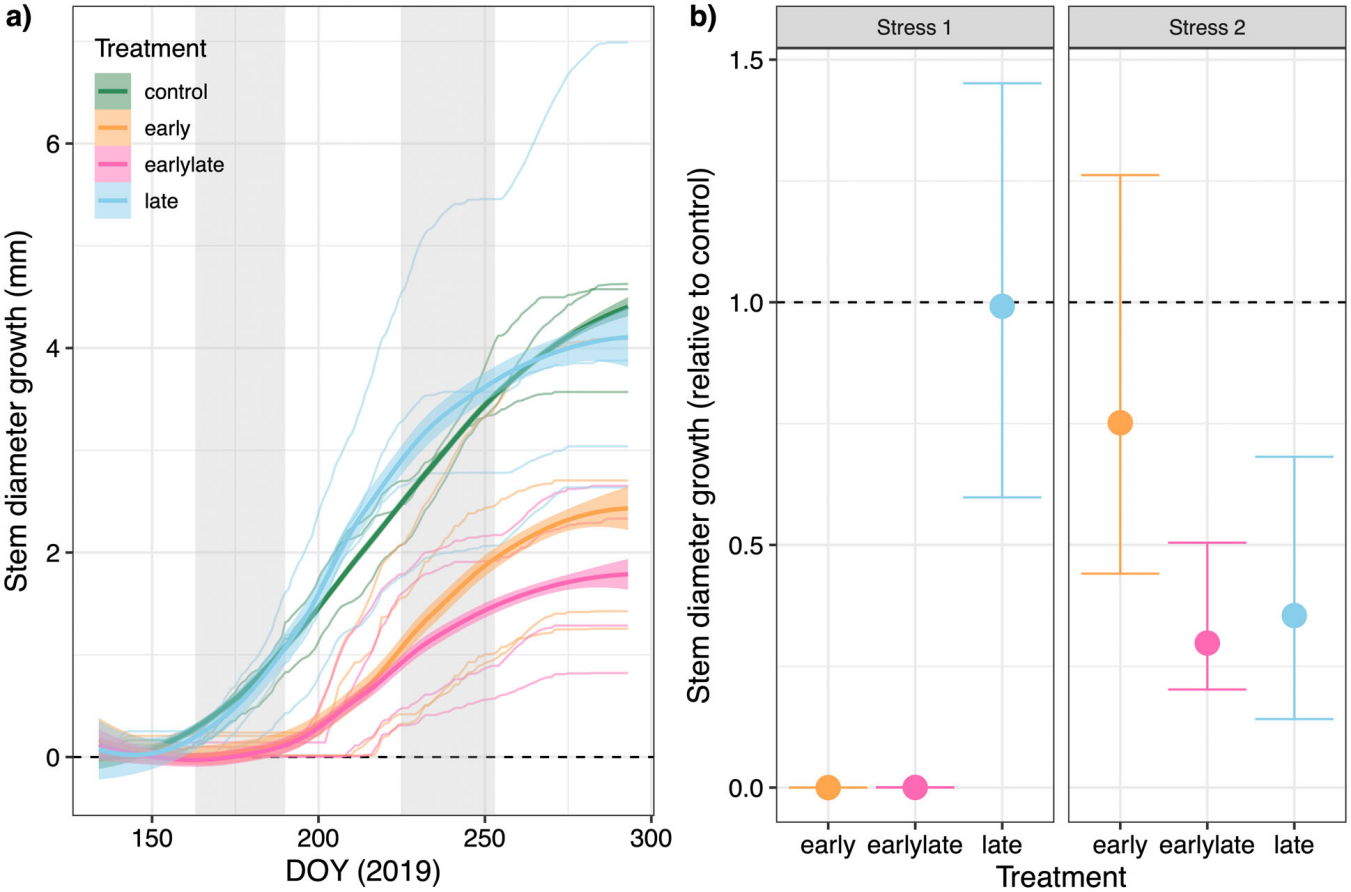
Impacts of differently timed hot‐drought stress on stem growth in Scots pine saplings. (a) Cumulative stem growth per individual tree is shown with thin lines, assuming periods of no growth (stem shrinkage) as zero. The overall treatment trend is illustrated using a LOESS smoothing function ±1 SD (line with shaded area). The two hot drought periods are indicated as grey shaded areas. (b) Relative stem diameter growth per treatment and stress period (Stress 1: DOY 163–191; Stress 2 DOY 225–253) compared to the control. Stem diameter growth for each of the two stress periods was calculated by subtracting stem diameter at the beginning of each stress period from stem diameter at the end of each stress period. Values shown represent relative mean difference to control, with a 95% CI derived from bootstrap analysis. The broken line represents control treatment, and non‐overlapping CI indicate significant differences.

Stem and needle growth exhibited distinct seasonality, particularly sensitive to early hot‐drought stress. The first, early‐stress period, lasting from 12 June to 9 July, when irrigation was stopped and SWC declined from ca. 35% to 22%. Alongside air temperature was stepwise increased to a maximum of 36.5 °C and a VPD of 3.5 kPa was reached (Fig. 1), while under ambient conditions maximum temperature was 26.5 °C with a VPD of 1.9 kPa. Concurrently, *Ψ*_leaf_ declined in response to the hot‐drought to a minimum of −1.4 MPa in the early‐stressed trees (Fig. 2a). The combination of moderate drought and high temperatures had two clear impacts on the growth trajectories of the saplings. First, expansion of new needles ceased ca. 2 weeks after the hot drought period was initiated (early: DOY 183 [174, 188]; early‐late: DOY 173 [170, 183]; Fig. 3) at a *Ψ*_leaf_ of ca. −1.2 MPa, while needle elongation under ambient conditions continued 28.8 ± 6.6 days longer (control: DOY 205 [202, 215], late: DOY 202 [195, 210]). Second, stem growth rates were delayed in the hot drought‐treated trees. While in the control, stem growth started at the beginning of June, in the early and early‐late trees clear signals of stem growth became apparent only after the end of the first hot‐drought stress by mid‐July (Fig. 4a). Albeit stem growth rates were partially recovering in early‐stressed trees, leaf expansion had irreversibly stopped.

During the late‐season hot drought (13 August to 10 September), the early‐late trees and the previously‐unstressed trees in the late treatment were exposed to a similar hot drought trajectory as during the early stress period in terms of temperature increment and duration, but with slightly higher maximum temperatures (38.2 °C) and VPD (4 kPa) to account for seasonal differences. Irrigation was stopped 7 days before the stepwise increase in temperature was started and SWC began to decline. During this late hot drought, *Ψ*_leaf_ did not decline below −1.1 MPa, indicating a lower overall stress intensity compared to the first period (Fig. 2a). Notably, trees that had undergone the first hot drought period maintained a significant higher *Ψ*_leaf_ than the trees in the late treatment (Tukey HSD *P* < 0.01 at DOY 253). This was also visible as a lesser tree water deficit in the early‐late compared to the late trees during the second stress phase, indicating decreased sensitivity to recurring drought (Fig. 2b). In agreement, with the occurrence of tree water deficit, growth rates stopped and, despite the overall low stress intensity, stem diameter growth during the second stress phase was reduced by 50–60% in the late and early‐late treatment (Fig. 4b). However, this did not reduce the absolute seasonal stem growth in the late trees, which did not differ from the control at the end of the experiment (Fig. 4a).

### Structural adjustments from early‐season hot‐drought stress

**Fig. 5:**
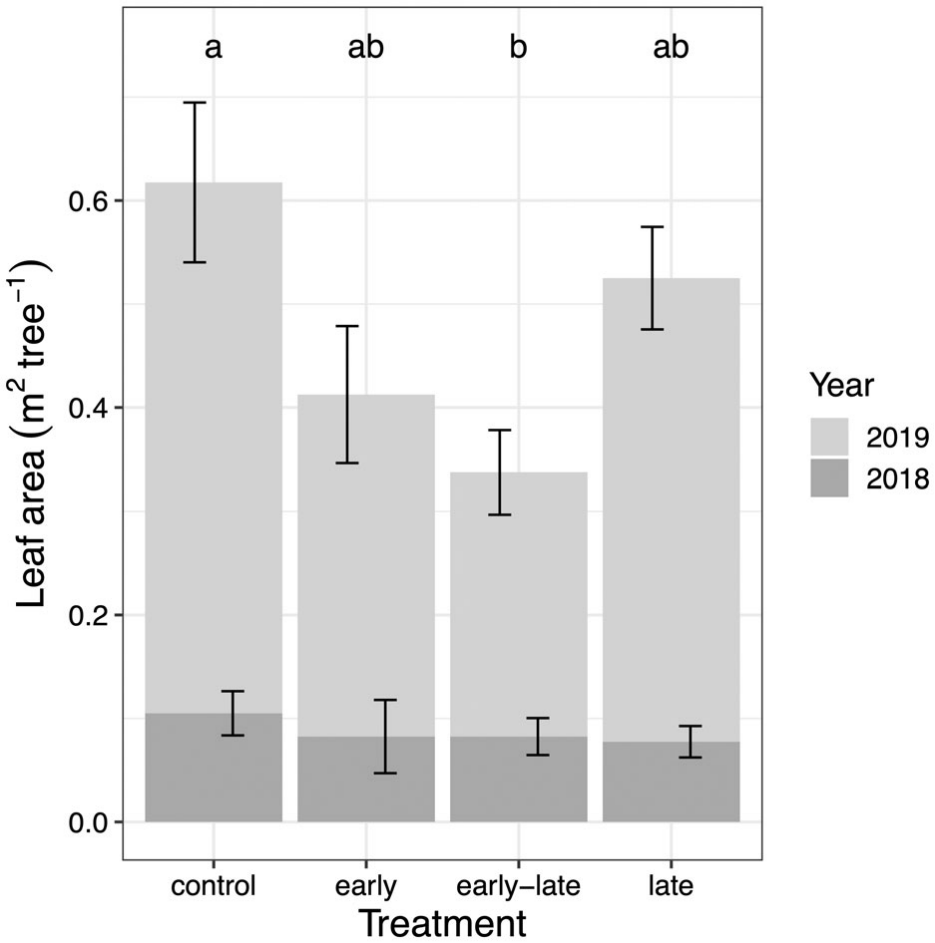
Leaf area of current‐year (2019) and 1‐year‐old (2018) needles in Scots pine at the end of the growing season. Treatment averages of total leaf area for current‐year and 1‐year‐old needles are shown (n = 4 per treatment). Error bars represent ±1 SE. Significant treatment differences of current‐year needles are given (Tukey HSD *P* < 0.05).

**Fig. 6:**
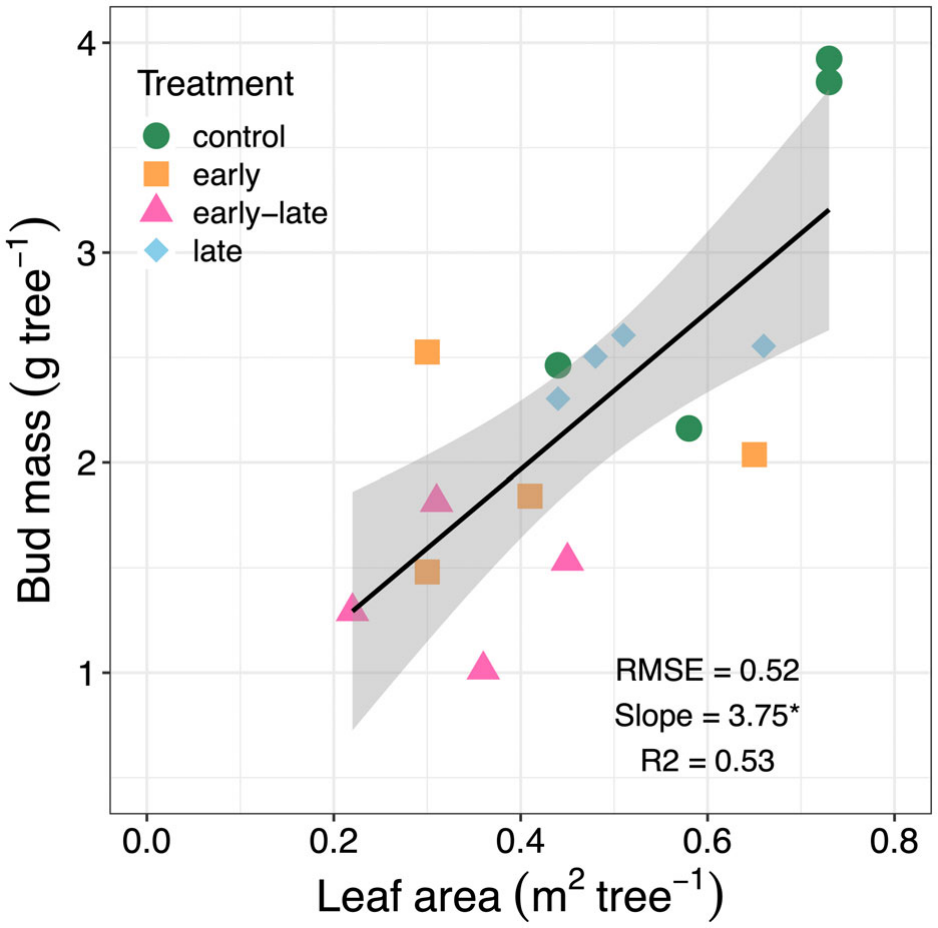
Relationship between bud mass and leaf area in Scots pine saplings at the end of the growing season. Tree‐specific data from the four treatments (n = 4) are depicted, and the solid black line indicates a linear relationship (*P* < 0.01), with shaded areas representing ±1SE of the fit. The coefficient of the slope was significantly different from zero, indicated by the asterix (*P* < 0.01).

Stress‐induced reductions in tree growth significantly affected end‐of‐season biomass and leaf area (Table 1 and Fig. 5). The earlier cessation of leaf expansion in response to the first stress period, resulted in needles that were approximately 2.7 cm shorter than those of the control trees. This reduction in needle length was also reflected in needle surface area, which was, on average, 30% lower in early‐stressed trees compared to control trees at the end of the experiment (F = 12.2, n = 18, *P* < 0.01; Table 1). Consequently, the reduction in needle area of current‐year needles led to a decrease in current‐year leaf area (Fig. 5), as well as a higher relative proportion of previous‐year needles to total leaf area in the early‐late trees (non‐overlapping CI). In contrast, leaf area of trees in the late treatment was not affected, as leaf development has ended before the second stress period. Additionally, bud mass at the end of the experiment was correlated with remaining leaf area (n = 15, F = 17.1, *P* < 0.01; Fig. 6), indicating a potential legacy effect of reduced leaf development on the following year's growth.

**Table 1:** Treatment impacts on end‐of‐season biomass in Scots pine saplings. Surface area per needle (n = 8 per treatment), leaf, root, and woody biomass were measured at the end of the experiment (n = 4 per treatment).

Treatment	needle area	leaf biomass	root biomass	wood biomass	bud mass	leaf‐to‐root
	cm^2^	g DW	g DW	g DW	g DW	cm^2^ gDW^−1^
early‐late	0.39 ± 0.03 b	52.7 ± 12.0 b	69.7 ± 7.7 a	91.8 ± 5.4 b	1.41 ± 0.17 b	55.7 ± 8.1 a
early	0.36 ± 0.02 b	66.9 ± 18.0 b	84.8 ± 12.2 a	110.3 ± 10.5 b	1.97 ± 0.21 b	56.0 ± 7.8 a
late	0.62 ± 0.03 a	104.0 ± 9.0 a	94.7 ± 14.1 a	127.3 ± 13.2 ab	2.49 ± 0.07 a	63.8 ± 8.6 a
control	0.57 ± 0.03 a	117.3 ± 13.5 a	106.9 ± 15.0 a	156.1 ± 15.6 a	3.09 ± 0.45 a	64.3 ± 3.6 a

*Note*: Results are mean ± SE. Differences between treatments were tested using ANOVA followed by a Tukey post‐hoc test. Letters show significant differences (*P* < 0.05).

Stress impacts on stem diameter increment were most pronounced in trees subjected to the early hot‐drought stress. This resulted in a ca. 50% lower stem diameter increment at the end of the experiment (Fig. 4), as well as reduced aboveground woody biomass in early‐stressed trees compared to control trees (Table 1). Albeit a significant growth reduction during the second stress phase (Fig. 4b), the effect of the late stress on end‐of‐season stem diameter growth and woody biomass appeared minimal.

### Legacies from early season stress alter tree transpiration

**Fig. 7:**
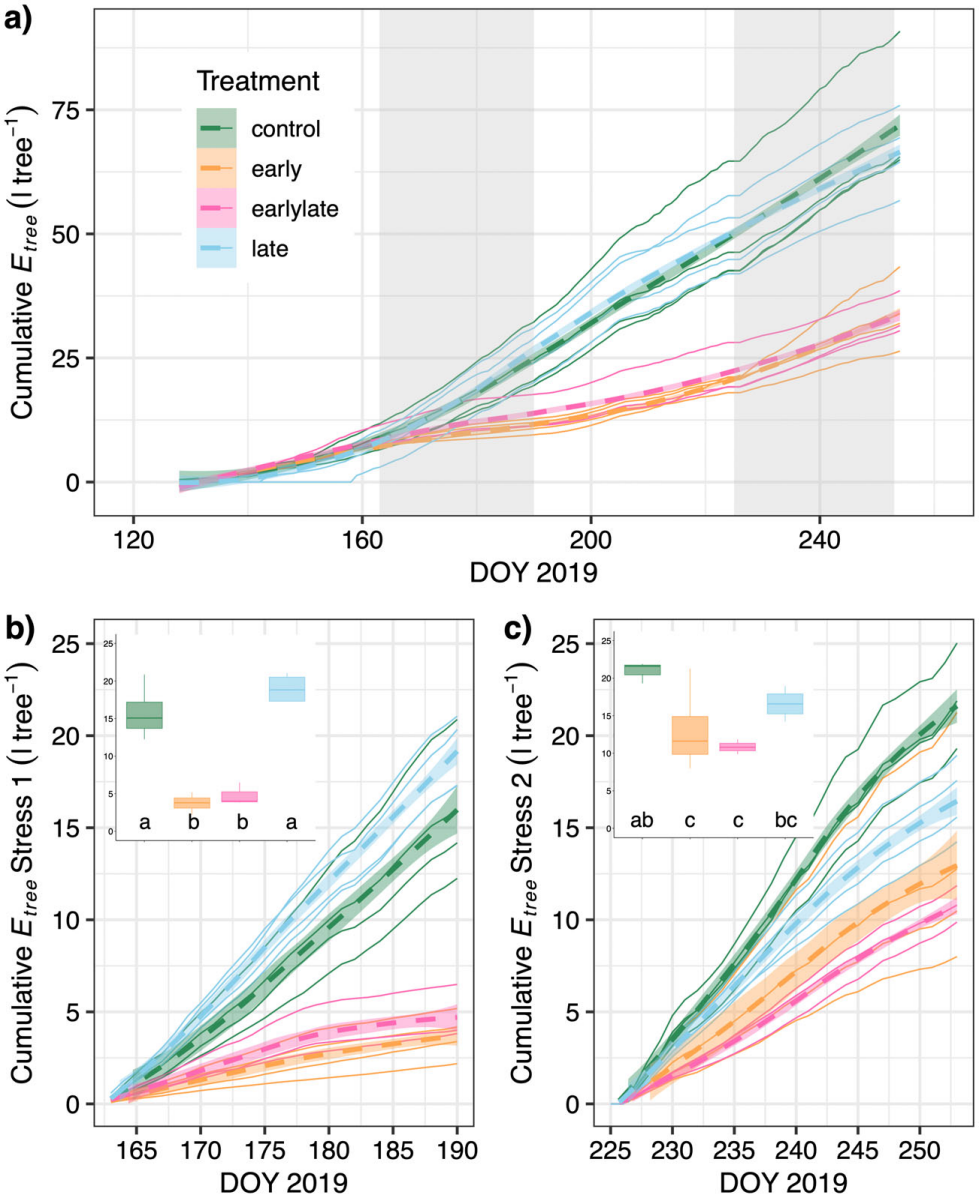
Dynamics of treatment‐specific cumulative tree transpiration (E_tree_, l tree^−1^) for Scots pine saplings. (a) Cumulative E_tree_ per tree and treatment (n = 3 for control, n = 4 for all other treatments) given as colored lines. Treatment average using a smoothing LOESS spline ±1SD as broken lines and shaded area. The two hot drought periods are depicted as grey shaded areas. (b, c) Drought‐specific cumulative transpiration during the first (b) and second (c) hot drought period. Small insets highlight treatment differences in cumulative E_tree_ over the stress period, shown as boxplots. Differences were tested using ANOVA followed by a post‐hoc Tukey HSD.

**Fig. 8:**
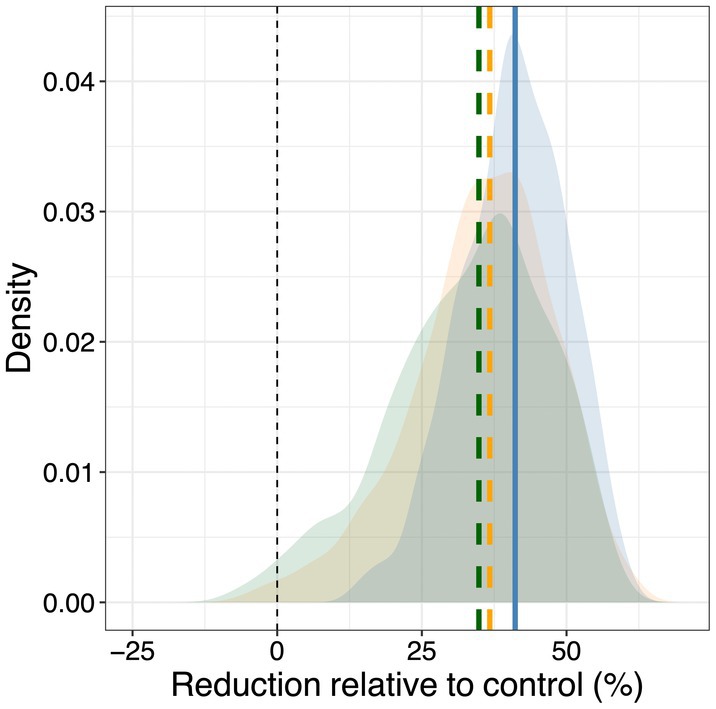
Comparison of leaf area and transpiration reduction at the end of the experiment (DOY 253–254). Distributions and means of relative decline in tree transpiration (blue), total leaf area (green) and current‐year leaf area (yellow) of the early hot drought treatment compared to the control. Frequency distributions and differences between the control and early hot‐drought treatment were obtained via bootstrapping.

Cumulative tree‐level transpiration (E_tree_) in the late and control trees showed a steep increase coinciding with leaf expansion (Fig. 7a). In contrast, E_tree_ in the early and early‐late trees showed a much lower increase in cumulative E_tree_ remaining ca. 70% below the control trees during the progression of the first hot drought period (Fig. 7b). Following release from the early‐season stress, cumulative E_tree_ in the early‐stressed trees recovered slightly and was on average ca. 40% below control trees at the end of the experiment, which coincides with the reduction in total leaf area of ca. 35% (Fig. 8). The second hot drought period resulted in a modest, but non‐significant, decline of cumulative E_tree_ in the late trees (Fig. 7c inset). E_tree_ in the early trees tended to be ca. 30% lower compared to the late trees, corresponding to the 30% reduction in needle area. This suggests that the early‐season stress period had lasting legacy effects on tree transpiration, related to the overall reductions in leaf area triggered by early hot‐drought stress.

## Discussion

### Tree growth responses to hot drought are sensitive to stress timing

The seasonal timing of a drought event is an often‐overlooked aspect in stress physiology, although it clearly matters in terms of stress impact. Our study demonstrates that Scots pine saplings are highly sensitive to hot‐drought stress during leaf expansion, where both leaf and stem growth ceased. Specifically, we observed that leaf expansion was highly sensitive to drought, with needle elongation and stem increment ceasing at a midday leaf water potential of ca. −1.1 MPa. This result aligns with previous studies showing that growth is a highly water sensitive process (Tang & Boyer 2002; Muller *et al*. 2011). Additionally, the simultaneous increase in VPD in our experiment during the hot drought, likely imposed further constraints on leaf expansion (Zweifel *et al*. 2021), as previously shown for pinon pine (Adams *et al*. 2015). This sensitivity is consistent with cambial cell turgor pressure being critical for cell elongation and differentiation, where even minor reductions in water availability can significantly impact growth (Lockhart 1965). While other studies, also have reported reduced needle growth in *Pinus* spp. in response to drought (Grill *et al*. 2004; Dobbertin *et al*. 2010; Adams *et al*. 2015), we could directly refer the decreased productivity to an earlier halt in needle elongation, not reversed upon stress release.

The observed morphological response aligns with findings from studies comparing non‐irrigated to irrigated *Pinus sylvestris* stands (Dobbertin *et al*. 2010; Zweifel *et al*. 2020) and other drought experiments in Mediterranean‐type forests (Misson *et al*. 2011; Adams *et al*. 2015). Our results indicate that this drought‐induced reduction in leaf area led to reduced productivity in the early and early‐late treatments, as seen in a lower overall biomass. In addition, current‐year needles typically have higher photosynthetic efficiency compared to older foliage (Warren 2006). Clearly, current‐year leaf area must be a dominant driver of productivity in these young seedlings, as previously observed in mature Scots pine trees (Zweifel *et al*. 2020). This confirms our first hypothesis that stress impacts early in the season cannot be fully compensated.

### Structural adjustments alter water‐use and drought sensitivity

Resolving the impacts of the two hot drought periods was challenged by a weaker stress signal from the late drought period. This might have been caused by more efficient use of soil water resources, as indicated by the apparent steeper decline in soil water content (Fig. 1c). Root growth was not restricted in our experiment because of the large pots used (~100 L soil volume), and trees were likely able to better access soil water reserves later in the season. However, as the root‐to‐shoot ratio did not differ between treatments, we expect that the water supply‐to‐demand ratio was not altered, which allows comparison of the relative stress responses of the saplings in the early‐late compared to the late treatment.

The reduced leaf area following the early stress phase lowered tree seasonal water use. Accordingly, we observed improved drought resistance of the early‐late compared to the (previously‐unstressed) late trees, reflected in significantly less negative water potentials together with less pronounced tree water deficits and a tendency for lower transpiration during the late‐season hot drought period. Re‐occurring droughts can result in improved stress resistance in Scots pine, as observed previously, particularly if structural adjustments occur (Seidel *et al*. 2019). Understanding whole‐tree water use and associated adjustments in leaf area is essential for a mechanistic understanding of dehydration processes under drought stress (Blackman *et al*. 2023). However, also other physiological factors, such as increased stomatal sensitivity, improved osmoregulation, or water‐use‐efficiency, could have emerged and altered responses to subsequent droughts, as for instance shown for relationships between stomatal conductance and VPD during soil drought in a previously irrigated Scots pine stand (Schönbeck *et al*. 2022). Nonetheless, it remains highly challenging to disentangle physiological from anatomical and structural adjustments as they are closely interlinked (*e.g*., Schönbeck *et al*. 2022; Gattmann *et al*. 2023). Most prominently in our study was a decrease in leaf development from early stress, which we show was closely related to reduced transpiration rates (Fig. 8). Thus, in agreement with others (Zweifel *et al*. 2020), we found that reductions in leaf area are a dominant factor in modifying the stress response in our seedlings. Therefore, we can largely confirm our second hypothesis, that reduced evaporative surface area alleviated midday leaf water potential declines during a later season stress period.

### Structural adjustments and drought legacies

Drought legacy effects on growth performance were further evidenced by reduced bud mass in trees subjected to early and early‐late stress. Previous studies have shown that bud mass decreases with increasing drought damage in beech saplings (Thomas *et al*. 2023). Typically, a positive correlation between bud mass and leaf development can be expected. For instance, in red pine, a direct link between bud size and shoot length has been reported (Kozlowski *et al*. 1973), and shoot length can be directly related to leaf area in conifers (Tanabe *et al*. 2022). Additionally, it has been demonstrated that longer shoots are produced following years with larger buds and ample water supply in pines (Clements 1970). Therefore, our results suggest that early‐season hot drought not only reduced the current year's leaf area but may also affect leaf area development in the subsequent year. This largely confirms our third hypothesis. Stress legacy responses can have significant consequences for growth performance, particularly in conifers (Galiano et al., 2011). For instance, an increase in leaf area in Scots pine from increasing water availability in a typical dry alpine valley during several years resulted in a larger productivity until 4 years after the irrigation had been stopped (Zweifel *et al*. 2020). Conversely, a stress‐induced reduction in leaf area in evergreen conifers with a slow leaf turnover can result in lower growth rates years after the stress event and may reduce individual competitiveness within a forest stand. Furthermore, low growth performance of conifers following release from drought and heat has been associated with reduced tree vitality, leading to a higher mortality risk (Sterck *et al*. 2024). However, a lower leaf area reduces the water demand of trees and thus might enhance the resilience to subsequent drought stress (Ziegler *et al*. 2024). This indicates that structural adjustments may result in both positive and negative legacies. In the short term a lower leaf area can improve stress performance, but over the longer term it may result in decreased competitiveness and tree vigor.

### Implications for modeling drought legacies

Changes in tree growth allocation from hot‐drought stress have the potential to affect forest carbon and water fluxes long after the stress event (Ruehr *et al*. 2019). These structural adjustments can influence tree responses to subsequent droughts (Sangüesa‐Barreda *et al*. 2023). Depending on the magnitude and direction of the adjustment, trees may become more susceptible and succumb if, for instance, limited resources are available for pest and pathogen defense (Sterck *et al*. 2024) or trees might adapt by reducing evaporative surface area and investing in improved hydraulic capacity, such as increased root production (Aaltonen *et al*. 2017). However, the sign and direction of such drought adjustments has only been marginally addressed in models yet. While there have been previous attempts to integrate morphological adjustments in response to drought into plant‐soil‐atmosphere models of little complexity (*e.g*., Magnani *et al*. 2002; Zweifel *et al*. 2020), these processes have so far been largely overlooked in more complex ecosystem‐scale models (but see Nadal‐Sala *et al*. 2024). Particularly in evergreen conifers, these may become critical as leaf lifespan is typically several years, increasing the risk of legacy responses and subsequent mortality (Sterck *et al*. 2024). Therefore, we advocate that model development should include stress‐induced anatomical adjustments to address legacy responses by including: (i) an explicit integration of soil and atmospheric drought‐induced cessation of growth, (ii) a description of drought damage of different tree compartments, and (iii) a delayed recovery of these impairments considering different turnover times of tissues. Integrating stress legacies into ecosystem models could capture both the positive impacts of hydraulic adjustments that reduce drought vulnerability, as well as the negative impacts of reduced growth and carbon uptake, potentially reducing the overall competitiveness within a forest stand. Moreover, such models that represent the different strategies of the drought avoidance–resistance strategy continuum (see Ziegler *et al*. 2024) will improve our abilities to simulate drought legacy impacts on forest ecosystems.

## Conclusion

To understand the long‐term impacts of drought and hot‐drought stress on trees, it is essential to analyze tree physiological responses to stressors occurring at various points throughout the growing season. Here we demonstrate that an early‐season hot drought led to limited needle elongation in Scots pine, resulting in legacy responses apparent in reduced seasonal tree water use, which improved resistance to subsequent stress when compared to previously unstressed trees. However, these structural adjustments resulted in persistent growth reductions, including reduced bud mass, which will have carry‐over effects into the next growing season. Particularly in evergreen conifers with a leaf lifespan of many years, this could potentially affect tree vitality and competitiveness at the stand level. Therefore, we recommend that future model development should incorporate structural adjustments of stress‐induced growth cessation to address the potential role of these adjustments in stress avoidance versus pre‐disposition to tree death, particularly in evergreen conifers with slow leaf turnover rates.

## AUTHOR CONTRIBUTIONS

N.K.R. designed and supervised the experimental study. D.N‐.S. analyzed the data with contributions from N.K.R.; N.K.R. and D.N‐.S. jointly wrote the manuscript draft.

## FUNDING INFORMATION

This study was supported by the German Research Foundation through its Emmy Noether Program (RU 1657/ 2–1). NKR acknowledges funding through the Initiative and Networking fund of the Helmholtz Association (W2/W3‐156). DN‐S was supported by the INFORMA EU project (Grant agreement: 101060309).

## CONFLICT OF INTEREST STATEMENT

The authors declare no conflicts of interest.

## Supporting information


**Figure S1** Growing season air temperature from a meteorological station of the German Weather Service in Weißenburg‐Emetzheim, Germany.
