# Supplementary material for: Legacies from early‐season hot drought: how growth cessation alters tree water dynamics and modifies stress responses in Scots pine
Source: Plant Biol (Stuttg). 2025 Jan 15;28(3):610–21. doi: 10.1111/plb.13760 (PMC13089592; doi:10.1111/plb.13760)
Supplement: Supplementary file 1 — Figure S1 Growing season air temperature from a meteorological station of the German Weather Service in Weißenburg‐Emetzheim, Germany. [file PLB-28-610-s001.pdf]

Supplementary

Title: Legacies from early-season hot drought: How growth cessation alters tree water dynamics and modifies stress responses in Scots pine

Authors: Nadine K. Ruehr; Daniel Nadal-Sala

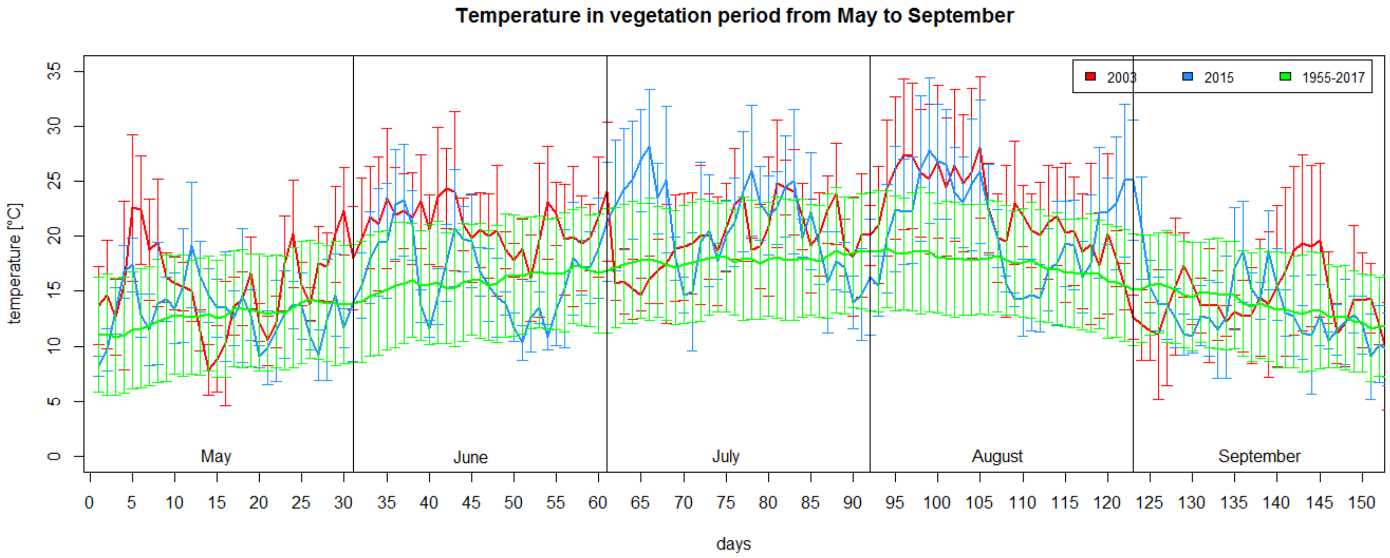

Figure S1 Growing season development of air temperature from a meteorological station of the German Weather Service in Weißenburg-Emetzheim, Germany. Data from 2003 and 2015 summer period, which were drier and hotter as the long-term average (1995–2017), were used as reference period for the adjustment of the temperature conditions during the hot-drought experiment. In this region Scots pine mortality has been observed following the hotter 2015 summer.
